# Common mental disorder and its association with academic performance among Debre Berhan University students, Ethiopia

**DOI:** 10.1186/s13033-017-0142-6

**Published:** 2017-05-03

**Authors:** Yohannes Gebreegziabhere Haile, Sisay Mulugeta Alemu, Tesfa Dejenie Habtewold

**Affiliations:** 1 Department of Nursing, College of Health Science, Debre Berhan University, 445, Debre Berhan, Ethiopia; 2 Mental Health and Psychosocial Support Program, International Medical Corps, Dolo Ado, Ethiopia; 3 Department of Epidemiology and Rob Giel Research Center, University of Groningen, University Medical Center Groningen, Groningen, The Netherlands

**Keywords:** Common mental disorder, Prevalence, Academic performance, Students, Ethiopia

## Abstract

**Background:**

Common mental disorder (CMD) is prevalent in industrialized and non-industrialized countries. The prevalence of CMD among university students was 28.8–44.7% and attributed to several risk factors, such as schooling. The aim of this study was to assess the prevalence and risk factors of CMD. In addition, the association between CMD and academic performance was tested.

**Methods:**

Institution based cross-sectional study was conducted with 422 students at Debre Berhan university from March to April 2015. CMD was the primary outcome variable whereas academic performance was the secondary outcome variable. Kessler psychological distress (K10) scale was used to assess CMD. Bivariate and multiple logistic regression analysis were performed for modeling the primary outcome variable; independent samples T test and linear regression analysis were carried out for modeling the secondary outcome variable. The strength of association was interpreted using odds ratio and regression coefficient (β) and decision on statistical significance was made at a p value of 0.05. Data were entered using EPI-data version 3.1 software and analyzed using the Statistical Package for the Social Sciences (SPSS) version 20.01 software.

**Results:**

The prevalence of CMD was 63.1%. Field of study (p = 0.008, OR = 0.2, 95% CI 0.04–0.61), worshiping (p = 0.04, OR = 1.8, 95% CI 1.02–3.35), insomnia (p < 0.001, OR = 3.8, 95% CI 2.21–6.57), alcohol drinking (p = 0.006, OR = 2.7, 95% CI 1.33–5.66), and headache (p = 0.02, OR = 2.1, 95% CI 1.10–3.86) were identified risk factors for CMD. The mean cumulative grade point average of students with CMD was lower by 0.02 compared to those without CMD, but not statistically significant (p = 0.70, β = −0.02, 95% CI −0.15 to 0.10). CMD explained only 0.8% (r^2^ = 0.008) of the difference in academic performance between students.

**Conclusions:**

At least three out of five students fulfilled CMD diagnostic criteria. The statistically significant risk factors were field of study, worshiping, insomnia, alcohol drinking, and headache. Moreover, there was no statistically significant association between CMD and academic performance. Undertaking integrated evidence-based intervention focusing on students with poor sleep quality, poor physical health, and who drink alcohol is essential if the present finding confirmed by a longitudinal study.

## Background

Mental health is a state of well-being in which every individual realizes his or her own potential, can cope with the normal stresses of life, can work productively and fruitfully, and able to contribute to her or his community [1]. Mental disorder is a syndrome characterized by a clinically significant disturbances in cognition, emotion regulation, or behavior accompanied by psychological, biological, or developmental processes dysfunction [2]. Mental disorders account for 14% of the global burden of disease; 75% of affected people are living in low-income countries [3]. In Ethiopia, mental disorder is the leading non-communicable disorder which made up 11% of the total burden of disease [4].

The social environment, academic norms, and psychosomatic reactions to diverse situation potentially affect the mental health of university students [5]. Research conducted by the National Alliance on Mental Illness in the US have shown that 25% college students had a diagnosable illness, 40% did not seek help, 80% felt overwhelmed by their responsibilities, and 50% had anxiety [6]. The American College Health Association survey report in 2010 also revealed that 45.6% of the students feeling hopeless and 30.7% feeling depressed [7].

The prevalence of mental distress, a non-specific form of altered mental health, in Ethiopian university students was found to be 21.6–49.1% [8–11]. The most consistent associated factors were a family history of mental illness, frequent conflicts with fellows, Khat chewing, worshiping, batch of students, field of study, level of training, and age [8–11]. In addition, another study reported that mental distress has been associated with the difficulty in making friends and dating, active sexual practice, income and stationary materials inadequacy, lack of adequate access to academic reference materials, lack of adequate access to sanitary and recreational facility, overcrowding, and worrying about personal safety [11].

Common mental disorder (CMD), also known as a minor psychiatric disorder, is characterized by insomnia, fatigue, irritability, forgetfulness, difficulty in concentration, and somatic complaints [12]. Globally, the prevalence of CMD was ranging from 7 to 50% [13–22]. Similarly, a meta-analysis of 174 studies concluded that the 1-year prevalence of CMD was 17.6% and the lifetime prevalence was 29.2%; both estimates were low in Asia and Sub-Saharan African countries [23]. Moreover, a cross-sectional survey in England, Wales and Scotland revealed that the prevalence of CMD was 17–31% [24, 25].

The prevalence of CMD was 28.8–44.7% among university students [26–30], 43.3% among community-based health agents [31], 50.1% among socio-educational agents [32], 22–42.6% among primary healthcare workers [33, 34], 22.3–34.5% among university employees [35], 30.2–50% among patients [36–39], 41.4% among pregnant women [40], 29.7–32.1% among elders [41, 42], 24% among physicians [43], and 6.7% among civil aviation pilots [44].

CMD has been associated with several factors. A systematic review of 115 studies in low and middle-income countries reported that CMD was strongly associated with poverty, education, food insecurity, housing, social class, socio-economic status, and financial stress [45]. Similarly, cross-sectional studies conducted in South America identified poverty, schooling, social inequality, low income, sex, age, employment status, inadequate body weight perception, tobacco smoking, violence, poor social support, sedentary behavior and body image dissatisfaction were risk factors of CMD [16, 17, 19, 20, 35, 36, 46, 47]. Moreover, Harpham et al. [18] found out gender, educational status, and violence were the risk factors of CMD. Weich et al. [24, 25] also concluded that high-income individuals to be more prone to CMD and vice versa.

Even though CMD is common in the general population, young people particularly university students are more susceptible [18, 46, 48]. A cross-sectional study with university students uncovered that the prevalence of CMD was 28.8–44.7% [26–30]. The risk factors were difficulty in making friends, poor self-evaluation of academic performance, thoughts of dropping out, sleep disorder, not owning a car, feeling overloaded, discrimination, limited physical activity, and perceived lack of emotional support [26–30]. A large cross-sectional web-based study conducted at the University of Newcastle found that nearly one-third of students reported at least one CMD [49]. The risk factors were financial stress, living alone, and low socioeconomic background [50, 51]. In addition, the prevalence of CMD among Dutch university medical students was 48–54% [52]. Another cross-sectional study conducted at the public university in Northeast Brazil reported that the prevalence of CMD was 33.7%; the risk factors were gender, lack of good expectations regarding the future, course as not a source of pleasure, and feeling emotionally tense [53].

The high public health burden of CMD has an impact on students interpersonal relationships and quality of life perhaps that affects their academic performance [27]. In addition, comparative data from the US have shown a significant link between high levels of psychological distress and low academic performance among college students [54]. Moreover, another earlier study discovered the association of mental illness and termination of university education, difficulty with time and resource management, and a decreased likelihood to seek academic assistance [55]. However, little is known about CMD in Sub-Saharan African countries particularly in Ethiopia. This gap pointed out the need to conduct further studies to measure the magnitude of mental health problem among university students and initiate culturally tailored evidence-based interventions [56]. Thus, the aim of this study was to assess the prevalence and risk factors of CMD. In addition, the association between CMD and academic performance was tested.

## Methods

### Study setting, design, and procedure

Institution based cross-sectional study was conducted at Debre Berhan University from March to April 2015. Debre Berhan University is located 130 km northeast from Addis Ababa, the capital city of Ethiopia. Currently, more than 14,000 regular, weekend, and summer program students were enrolled in 35 departments [57]. Undergraduate students who were enrolled in 2014/2015 full-time study, capable of independent communication, and provided informed written consent were included. All students were selected by proportionate stratified random sampling method. First, stratum was created using each discipline/college as a cluster. Second, students list was obtained from the academic record office. Third, based on the calculated sample size, the required number of students were allocated to each college proportional to the total number of students enrolled in the corresponding college. Fourth, simple random sampling method was used to reach the individual student. The sample size was determined using single population proportion formula considering the following assumptions: the prevalence of CMD was 50%, the margin of error was 5%, and confidence level was 95%. After adjustment for 10% non-response rate, the final sample size was 422.

#### Variables

Common mental disorder (CMD) was the primary outcome variable. CMD was diagnosed if Kessler psychological distress (K10) scale score was ≥7. Academic performance was the secondary outcome variable. Self-reported cumulative grade point average (CGPA) was used as a proxy measure of academic performance. Socio-demographic characteristics, substance use habit, and physical illness symptoms were the explanatory variables. Insomnia was assessed using the Pittsburgh Sleep Quality Index (PSQI) standard instrument with a global score cut-off value of >5 for cases. Worshipping was defined as any reported religious practice performed by students irrespective of their religion.

#### Data collection and instrument

The data were collected from nine disciplines using a structured self-administered questionnaire. The questionnaire had four different sub-sections: section one-sociodemographic characteristics; section two-K10 scale; section three-substance use habit; and section four-physical and psychological symptoms. K10 scale is a 10-item questionnaire that a person rating the 30 days anxiety and depressive symptoms experience in a five-level Likert scale. K10 scale has already been validated in Ethiopia by Tesfaye et al. [58] and yielded an excellent internal consistency of 0.93, sensitivity of 84.2%, and specificity of 77.8% at a cut-off point of 6/7. Thus, it was reasonable to use for this study population. The data were collected by 35 trained university instructors. Supervisors provided all relevant support when necessary.

#### Instrument reliability analysis

The K10 scale items had an excellent reliability for this study population. The interclass correlation (Cronbach’s Alpha) of items was 0.900 with Cronbach’s Alpha based on standardized items value of 0.901. Two-way mixed effects model and average consistency measure were used to measure the intraclass correlation of items, which was 0.9 (95% CI 0.88, 0.91).

#### Data processing and analysis

Before analysis, the data passed through stringent quality control process and inconsistencies, outliers, and missing values were checked using frequency distribution. Multiple imputations (5×) was done assuming the data values were missing at random. First, all explanatory variables were fitted step-by-step to the bivariate logistic regression model. Then, multiple logistic regression model analysis was done. Finally, the independent risk factors were selected if the p value was ≤0.05. The strength of association was determined using odds ratios with 95% confidence interval. Independent Samples T test was used to test the group difference in academic performance related to CMD while linear regression analysis was done to investigate the association between CMD and academic performance and estimate the explained variance. The effect of CMD on academic performance was interpreted using regression coefficient (β). Finally, the results were presented using charts and tables. EPI-data version 3.1 software was used for data entry, variable coding, and cleaning while SPSS version 20.01 software was used for analysis. The study was adherent to the Strengthening the Reporting of Observational Studies in Epidemiology (STROBE) statement [59].

## Results

### Missing data

In this study, even if some data were missing for the independent variables, no data was missing for dependent variables (K10 scale). Analysis of patterns of missing values revealed that a total of 28 variables had at least one missing value and a total of 249 students didn’t reply at least for one variable. Overall, 4% of the total sample data was missing. Since only a small percentage of the data was missing and the sample size was small, multiple imputation was done to handle missingness.

### Socio-demographic characteristics

Of the 422 students invited, 388 (91.9%) completed the self-administered questionnaire and 78.4% (304/388) were male. The mean age of students was 22.13 year (SD = 2.12). As illustrated in Table 1, 29.6% (115/388) of students were fifth year, 72.4% (281/388) were Amhara, 76.5% (297/388) were single, and 85.1% (330/388) were Orthodox Christian.

**Table 1:** Socio-demographic characteristics of Debre Berhan University students, April 2015

Characteristics	Frequency		K10 score	
	n	%	Mean	SD
Sex				
Male	304	78.4	10.45	7.94
Female	84	21.6	10.59	8.88
Field of study				
Natural and computational science	51	13.1	11.45	8.63
Agricultural science	24	6.2	9.75	8.00
Business and economics	59	15.2	11.9	7.47
Computer science and IT	25	6.4	12.76	10.11
Engineering	171	44.1	9.48	7.57
Humanity and social science	36	9.3	11.38	9.19
Others^a^	22	5.7	8.91	8.54
Educational level				
1st year	89	22.9	11.17	8.65
2nd year	100	25.8	10.01	7.98
3rd year	70	18.0	11.16	8.42
4th year	14	3.6	10.50	7.08
5th year	115	29.6	9.90	7.90
Ethnicity				
Amhara	281	72.4	11.00	8.50
Oromo	47	12.1	8.91	6.81
Others^b^	60	15.5	9.24	7.12
Religion				
Orthodox Christian	330	85.1	10.73	8.28
Protestant	31	8.0	10.16	8.69
Others^c^	27	7.0	7.69	4.93
Worshiping				
Frequently (daily)	171	44.1	9.86	8.52
Less frequently	197	50.8	11.17	7.91
Never	20	5.2	8.95	6.62
Marital status				
Single	297	76.5	10.48	7.97
In relationship	65	16.8	11.35	8.94
Others^d^	26	6.7	8.24	7.99
Additional work				
Yes^e^	30	7.7	11.66	8.39
No	358	92.3	10.38	8.13

N = 388

^a^Law, Health Science and Medicine

^b^Tigray, Gurage, Agaw, Sidama, Afar, Awi, Wolayita, Gamo, Silte, Hadiya, Konso and Gedeo

^c^Catholic, Adventist and Apostolic church

^d^Divorce and married

^e^Wood work, university police, daily laborer, helping family, pool keeping, guider, driver, farming, religious education, construction forman, any kind of work, merchant and teacher

### Substance use habit

As shown below in Table 2, 95.4% (370/388) of students did not smoke cigarettes, 92.3% (358/388) did not chew Khat, and 25.8% (100/388) drank alcohol less than once per month.

**Table 2:** Substance use habit of Debre Berhan University students, April 2015

Characteristics	Frequency		K10 score	
	n	%	Mean	SD
Smoking cigarettes				
No	370	95.4	10.42	8.02
Yes (any frequency)	18	4.6	11.62	10.62
Chewing Khat				
No	358	92.3	10.56	8.15
Yes (any frequency)	30	7.7	9.44	8.14
Drinking alcohol				
Never	222	57.2	9.55	8.23
Less than once per month	100	25.8	11.65	7.60
More than once per month	66	17.0	11.84	8.39

N = 388

### Physical and psychological complaints

During the last month, 38.9% (151/388) of students had a headache and 36.3% (138/388) had a fever. In addition, 61.6% (239/388) of students were insomniacs (Table 3).

**Table 3:** Physical and psychological complaints of Debre Berhan University students, April 2015

Characteristics	Frequency		K10 score	
	n	%	Mean	SD
Headache				
Yes	151	38.9	13.50	7.91
No	237	61.1	8.53	7.69
Back pain				
Yes	72	18.6	14.94	8.55
No	316	81.4	9.42	7.68
Fever				
Yes	138	36.3	12.94	7.95
No	240	63.7	9.04	7.91
Other complaints^a^				
Yes	78	20.1	14.28	7.61
No	310	79.9	9.51	8.01
Suicidal thought				
Yes	22	5.7	16.54	9.51
No	366	94.3	10.11	7.91
Insomnia				
No	149	38.4	6.70	5.96
Yes	239	61.6	12.83	8.44

N = 388

^a^Pain, respiratory disease, gastrointestinal disease, renal disease, and other (anemia, hypertension, fungal infection of the hair, heart problem, ear problem, sadness, hopelessness, fatigue, lack of interest, stress, anxiousness, happiness, and depression)

### Kessler psychological distress (K10) score and prevalence of common mental disorder (CMD)

**Fig. 1:**
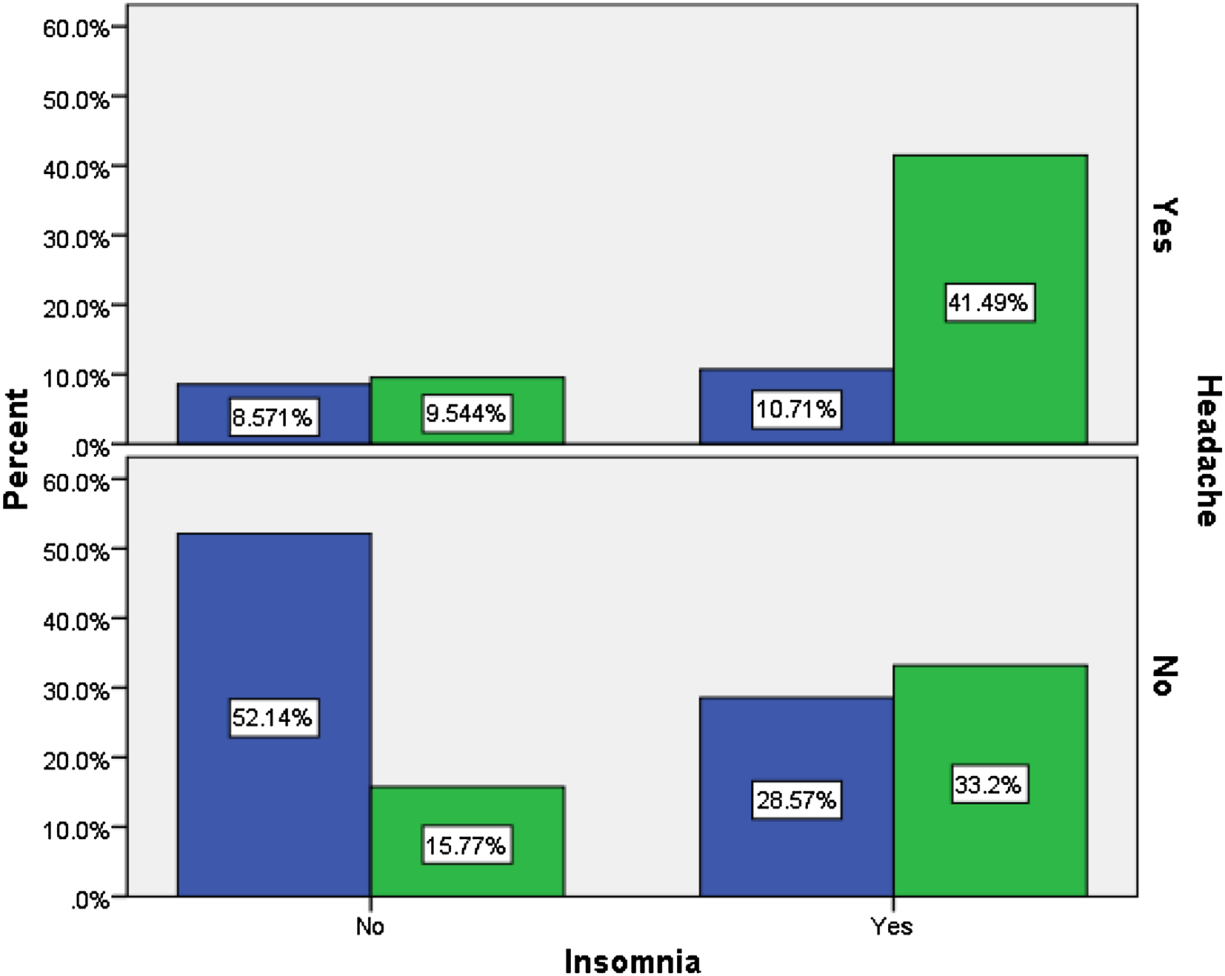
CMD by headache and insomnia at Debre Berhan University, April 2015. *Blue* no, *green* yes

The mean of K10 scale score was 10.48 (SD = 8.14) with the maximum score of 39. The prevalence of CMD was 63.1% (245/388). In addition, Fig. 1 gives a graphical description of the relationship between CMD, headache, and insomnia.

#### Risk factors of CMD

As presented in Table 4, field of study and worshipping were independent socio-demographic risk factors for CMD. Law and Health Science and Medicine students were significantly less likely (80%) develop CMD compared to Natural and Computational Science students (p = 0.008, OR = 0.2, 95% CI 0.04–0.61). Students who worshiped less frequently were 1.8 times more likely develop CMD compared to those students who worshiped daily (p = 0.04, OR = 1.8, 95% CI 1.02–3.35).

**Table 4:** Association between CMD and socio-demographic characteristics, April 2015

Variables	CMD (K10 score ≥7)		Bivariate regression model		Multiple regression model	
	No n (%)	Yes n (%)	p value	OR (95% CI)	p value	OR (95% CI)
Sex						
Male	110 (36.2)	194 (63.8)				
Female	33 (39.3)	51 (60.7)	0.63	0.9 (0.54, 1.45)	0.47	1.3 (0.64, 2.58)
Field of study						
Natural and computational science	14 (27.5)	37 (72.5)				
Agricultural science	7 (29.2)	17 (70.8)	0.88	1.0 (0.31, 2.69)	0.34	0.5 (0.14, 1.97)
Business and economics	16 (27.1)	43 (72.9)	0.97	1.0 (0.44, 2.36)	0.15	0.5 (0.17, 1.33)
Computer science and IT	8 (32.0)	17 (68.0)	0.68	0.8 (0.28, 2.28)	0.13	0.4 (0.09, 1.37)
Engineering	73 (42.7)	98 (57.3)	0.05	0.5 (0.26, 1.01)	0.05	0.4 (0.12, 0.99)
Humanity and social science	13 (36.1)	23 (63.9)	0.39	0.7 (0.27, 1.67)	0.07	0.4 (0.11, 1.07)
Others^a^	12 (54.5)	10 (45.5)	0.03	0.3 (0.11, 0.89)	0.01	0.2 (0.04, 0.61)
Batch						
1st year	27 (30.3)	62 (69.7)	0.12	1.6 (0.88, 2.85)	0.57	0.7 (0.22, 2.35)
2nd year	41 (41.0)	59 (59.0)	0.98	1.0 (0.58, 1.72)	0.27	0.6 (0.23, 1.50)
3rd year	24 (34.3)	46 (65.7)	0.37	1.3 (0.71, 2.46)	0.67	0.8 (0.27, 2.32)
4th year	4 (28.6)	10 (71.4)	0.39	1.7 (0.51, 5.84)	0.72	1.4 (0.24, 8.01)
5th year	47 (40.9)	68 (59.1)				
Ethnicity						
Amhara	95 (33.8)	186 (66.2)				
Oromo	23 (48.9)	24 (51.1)	0.05	0.5 (0.29, 1.01)	0.08	0.5 (0.22, 1.09)
Others^b^	25 (41.7)	35 (58.3)	0.32	0.7 (0.42, 1.33)	0.30	0.6 (0.29, 1.47)
Religion						
Orthodox Christian	118 (35.8)	212 (64.2)				
Protestant	15 (48.4)	16 (51.6)	0.14	0.6 (0.27, 1.20)	0.83	0.9 (0.31, 2.56)
Others^c^	10 (37.0)	17 (63.0)	0.86	0.9 (0.40, 2.14)	0.10	2.6 (0.82, 8.54)
Worshiping						
Frequently (daily)	73 (42.7)	98 (57.3)				
Less frequently	63 (32.0)	134 (68.0)	0.04	1.5 (1.02, 2.39)	0.04	1.8 (1.02, 3.35)
Never	7 (35.0)	13 (65.0)	0.52	1.4 (0.52, 3.60)	0.91	0.9 (0.28, 3.13)
Marital status						
Single	108 (36.4)	189 (63.6)				
In relationship	22 (33.8)	43 (66.2)	0.49	1.2 (0.68, 2.21)	0.84	1.1 (0.50, 2.31)
Others^d^	13 (50.0)	13 (50.0)	0.17	0.6 (0.25, 1.27)	0.31	1.8 (0.58, 5.35)
Additional work						
Yes^e^	9 (30.0)	21 (70.0)	0.37	1.5 (0.64, 3.38)	0.48	1.5 (0.50, 4.30)
No	134 (37.4)	224 (62.6)				
Age			0.10	1.0 (0.81, 1.02)	0.16	0.9 (0.72, 1.06)

^a^Law, Health Science and Medicine

^b^Tigray, Gurage, Agaw, Sidama, Afar, Awi, Wolayita, Gamo, Silte, Hadiya, Konso and Gedeo

^c^Catholic, Adventist and Apostolic church

^d^Divorce and married

^e^Wood work, university police, daily laborer, helping family, pool keeping, guider, driver, farming, religious education, construction forman, any kind of work, merchant and teacher

Furthermore, insomnia, alcohol drinking, and headache were strongly associated risk factors of CMD. Insomniac students were 3.8 times more likely develop CMD compared to non-insomniacs (p < 0.001, OR = 3.8, 95% CI 2.21–6.57). Students who drink alcohol less than once per month were 2.7 times more likely develop CMD compared to students never drink alcohol (p = 0.006, OR = 2.7, 95% CI 1.33–5.66). Moreover, students who had headache were 2.1 times more likely develop CMD compared to those who had no headache (Table 5).

**Table 5:** Association between CMD and substance use habit and health complaints, April 2015

Variables	CMD (K10 score ≥7)		Bivariate regression model		Multiple regression model	
	No n (%)	Yes n (%)	p value	OR (95% CI)	p value	OR (95% CI)
Cigarettes smoking						
No	136 (36.8)	234 (63.2)				
Yes (with any frequency)	7 (38.9)	11 (61.1)	0.83	0.9 (0.34, 2.38)	0.95	1.1 (0.23, 4.85)
Khat chewing						
No	130 (36.3)	228 (63.7)				
Yes (with any frequency)	13 (43.3)	17 (56.7)	0.42	0.7 (0.33, 1.58)	0.39	0.6 (0.15, 2.12)
Alcohol drinking						
Never	98 (44.1)	124 (55.9)				
Less than once per month	24 (24.0)	76 (76.0)	0.001	2.5 (1.49, 4.35)	0.01	2.7 (1.33, 5.66)
More than once per month	21 (31.8)	45 (68.2)	0.05	1.8 (0.99, 3.23)	0.04	2.4 (1.02, 5.86)
Headache						
Yes	27 (17.9)	124 (82.1)	<0.001	4.4 (2.70, 7.23)	0.02	2.1 (1.10, 3.86)
No	116 (48.9)	121 (51.1)				
Back pain						
Yes	11 (15.3)	61 (84.7)	<0.001	3.6 (1.85, 7.15)	0.05	2.3 (1.00, 5.19)
No	132 (41.8)	184 (58.2)				
Fever						
Yes	29 (20.6)	112 (79.4)	<0.001	3.2 (1.96, 5.11)	0.08	1.7 (0.92, 3.14)
No	114 (46.2)	133 (53.8)				
Other complaints^a^						
Yes	9 (11.5)	69 (88.5)	<0.001	5.5 (2.65, 11.24)	0.002	3.9 (1.67, 9.07)
No	134 (43.2)	176 (56.8)				
Suicidal thought						
Yes	4 (18.2)	18 (81.8)	0.07	2.8 (0.92, 8.41)	0.35	0.5 (0.13, 2.06)
No	139 (38.0)	227 (62.0)				
Insomnia						
No	86 (57.7)	63 (42.3)				
Yes	57 (23.8)	182 (76.2)	<0.001	4.4 (2.81, 6.77)	<0.001	3.8 (2.21, 6.57)

^a^Pain, respiratory disease, gastrointestinal disease, renal disease, and other (anemia, hypertension, fungal infection of the hair, heart problem, ear problem, sadness, hopelessness, fatigue, lack of interest, stress, anxiousness, happiness, and depression)

### CMD and academic performance

The mean CGPA was 3.11 (SD = 0.42) with a maximum of 4.00 and a minimum of 1.73 points. Since the distribution of CGPA was normal and all assumptions of linear regression were fulfilled, linear regression analysis was used to test the association between CMD and academic performance. CMD explained only 0.8% (*r* ^*2*^ = 0.008) of CGPA variability between students. The mean CGPA of students with CMD was lower by 0.02 compared to those without CMD. However, it was not significant (p = 0.70, β = −0.02, 95% CI = −0.15–0.10).

## Discussion

In this study, the prevalence of CMD was 63.1%. This finding was in line with the previous study report in the Netherland university students [52]. On the other hand, it was approximately two to three times the prevalence of CMD in Ethiopian university students [60], Chilean university students [61], and Peruvian college students [62]. Moreover, the current study finding was higher than the study report by Silva et al. [63], Volcan et al. [64], and Haregu et al. [65].

In the present study, field of study was one of the risk factors for CMD; Law and Health Science and Medicine students had less odds of CMD compared to Natural and Computational Science students. The possible explanation was that Natural and Computational Science students study a hard science, such as mathematics, physics which is usually stressful and academically demanding to students. In the contrary, recent studies with university students concluded that the risk of CMD was high among Health Science and Medicine students [26–29]. The present study also uncovered that CMD was significantly associated with worshipping; students who worshiped less frequently were 1.8 times more likely develop CMD compared to those students who worshiped daily. The possible explanation was that worshipping helps to relieve stress and become optimistic about any negative life circumstances. This finding was in congruence with the study report in Brazil college students where low and moderate spiritual wellbeing showed a doubled risk of CMD [64].

Another important significantly associated risk factor was insomnia; insomniac students were 3.8 times more likely develop CMD compared to non-insomniacs. This finding was consistent with other previous studies report by Hidalgo et al. [66] among Brazilian medical students, Byrd et al. [60] among Ethiopian undergraduate students, Concepcion et al. [61] among Chilean university students, Rose et al. [62] among Peruvian college students, and Haregu et al. [65] among Thai college students. Furthermore, this study showed that alcohol drinking significantly increased the risk of CMD; students who drink alcohol less than once per month were 2.7 times more likely develop CMD compared to students never drink alcohol. This finding was similar to the study report by Byrd et al. [60] among Ethiopian undergraduate students, but on the other hand, the study conducted among Chilean [61], Peruvian [62], and Thai [65] university students did not confirm this significant association.

Finally, the current study sought the association between CMD and academic performance; the mean CGPA of students with CMD was lower by 0.02 compared to those without CMD though insignificant. This does not imply CMD has no relevant effect on students' academic performance. Therefore, this non-significant result might be due to two reasons. Primarily, this study had used CGPA which might be distorted by previous semester or year grade. This justification was supported by the finding that more than 75% of the students in this study were the second year and above. Secondly, the data was collected from students who actively attending their education perhaps their coping mechanism is good and academically competent. Nevertheless, the previous studies reported that CMD determine academic performance [67, 68].

Generally, heterogeneities have seen on the prevalence and risk factors of CMD and the association between CMD and academic performance as well. This might be due to the following reasons. First, Kessler psychological distress (K10) scale was used in the present study whereas all previously reviewed studies were used General Health Questionnaire (GHQ-12) and Self-Report Questionnaire (SRQ-20) to assess mental health status. Second, the current data was collected during examination week perhaps anticipated stress increased K10 scale score. Third, most of the previous studies were conducted only with medical students; however, this study recruited students from nine disciplines. Fourth, the current study assessed only the 30 days mental health status.

In one hand, by 2030 World Health Organization (WHO) targeted to reduce non-communicable diseases related premature mortality by one-third through prevention, treatment, and promotion of  mental health [69]. On the other hand, contemporary epidemiological studies in high and low-income countries found a significant association between mental disorders and educational achievement during tertiary education [67, 68]. Therefore, developing (inter)national mental health strategy has a pivotal role to achieve WHO health goal and improve students’ academic accomplishment. For the successful realization of the strategy, academic institutions and researchers should provide updated evidence-based information for delivering the most cost effective culturally tailored care.

This study has several implications to develop a universal culturally appropriate screening tool for the students who are at risk of CMD, serve as a baseline for future studies, and provide important evidence to plan need-based interventions for students with CMD. Meanwhile, the universal screening activity is not time-consuming, as a result, it can be integrated into a student clinic at the university. Furthermore, this study will be used as a baseline evidence for future mental heal care planning and intervention.

K10 scale, a standardized validated tool, was used to assess CMD. To the best of our knowledge, this study was the first that assessed CMD using K10 scale in university students. Moreover, a large number of students were recruited from nine disciplines. However, this study had several limitations. First, self-administered data were used that might added recall bias and socially desirability bias. Second, the cross-sectional nature of the study does not allow attribution of causality. Hence, the prevalence of CMD that was reported may not be exclusive to the situation on university alone. Finally, since our study was conducted only in one institution it might limit the external validity of results. However, this limitation was perhaps compensated by the inclusion of students from different ethnicity and socioeconomic group.

## Conclusions

At least three out of five students fulfilled CMD diagnostic criteria. The statistically significant risk factors were field of study, worshiping, insomnia, alcohol drinking, and headache. Moreover, this study concluded that there was no statistically significant association between CMD and academic performance. Undertaking integrated evidence-based intervention focusing on students with poor sleep quality, poor physical health, and who drank alcohol is essential if the present finding confirmed by a longitudinal study. The high prevalence of CMD suggests that immediate preventive and curative measures should be implemented, such as the setting up of psycho-pedagogic support and counseling services to build students resilience [53]. Life skill training is also required to openly discuss and actively address the problems during university education [55]. Furthermore, the students should be taught different stress management techniques to improve their ability to cope with a demanding professional course, such as hard science courses [70]. In order to have a better understanding of students’ mental health trajectory and educational achievement, longitudinal and interventional study should be conducted.
